# Contraception Use among Women Visiting Outpatient Department of Gynaecology in a Tertiary Care Centre: A Descriptive Cross-sectional Study

**DOI:** 10.31729/jnma.7965

**Published:** 2023-02-28

**Authors:** Meenu Maharjan, Bibechan Thapa, Heera Tuladhar, Yam Prasad Dwa, Sunita Bhandari, Smrity Maskey Pradhan, Minaxi Thakur, Manisha Bajracharya, Santosh Sharma

**Affiliations:** 1 Department of Gynaecology and Obstetrics, KIST Medical College and Teaching Hospital, Mahalaxmi, Lalitpur, Nepal; 2 Department of Emergency Medicine and Inpatient care, Nepal National Hospital, Kalanki, Kathmandu, Nepal

**Keywords:** *contraception*, *family planning*, *prevalence*, *women*

## Abstract

**Introduction:**

Family planning services can bring a wide range of benefits to women, their families and society as a whole. Many women of reproductive age have little or incorrect information about family planning methods. Even when they know some methods of contraceptives, they don't know the availability or how to use them properly. The aim of this study is to find out the prevalence of contraception use among women visiting the outpatient department of gynaecology of a tertiary care centre.

**Methods:**

A descriptive cross-sectional study was conducted among women visiting the gynaecological outpatient department from 10 April 2021 to 10 April 2022 after taking ethical approval from the Institutional review committee (Reference number: 2079/80-03). Women aged 18 to 49 years visiting during the study period were included and pregnant, postmenopausal and unmarried women were excluded from the study. Data was collected from one-to-one interviews. A convenience sampling method was used. Point estimate and 95% confidence interval were calculated.

**Results:**

Out of 208 patients, 146 (70.19%) (63.97-76.41, 95% Confidence Interval) women were currently using contraceptives. Short-acting reversible contraception was used by 97 (66.44%) and long-acting reversible contraception was used by 23 (15.75%). A total of 21 (14.38%) women used permanent sterilisation. The most commonly used contraceptive device was Depo Provera, 43 (29.45%) followed by condoms, 29 (19.86%).

**Conclusions:**

The prevalence of contraception use is lower than the other studies done in similar settings. Therefore, contraception promotion programs have to be encouraged to promote the efficient use of contraception.

## INTRODUCTION

Family planning (FP) services can bring a wide range of benefits to women, their families and society as a whole.^1^ FP can help in reducing maternal mortality by decreasing the number of unwanted pregnancies and risky abortions, and the proportion of births at high risk. It has been estimated that fulfilling women's unmet need for modern contraceptives would save about 140,000 to 150,000 maternal lives annually.^2,3^

Failure to plan a pregnancy can adversely affect the health of the family as a whole.^4^ Even when women know some methods of contraceptives, they do not know the availability or how to use them properly.^5^ Knowledge, attitude and practices towards family planning are the basic fundamentals of achieving the goals and targets of family planning.^6^

The aim of this study was to find out the prevalence of contraception use among women visiting the outpatient department of gynaecology of a tertiary care centre.

## METHODS

A descriptive cross-sectional study was carried out among women attending the outpatient Department of Gynaecology from 10 April 2021 to 10 April 2022 at KIST Medical College and Teaching Hospital. Ethical approval was taken from the Institutional Review Committee of the same institute (Reference number: 2079/80-03). Women aged 15 to 49 years coming to the outpatient department (OPD) for any gynaecological problem were included in the study irrespective of their complaints and diagnosis. Postmenopausal, unmarried or pregnant women were excluded from the study. Convenience sampling was done. The sample size was calculated by using the following formula:


$$\begin{array}{ll} \text{n} & ={\text{Z}}^{2}\times \frac{\text{p}\times \text{q}}{{\text{e}}^{\text{2}}}\\ & =1.96^{2}\times \frac{0.50\times 0.50}{0.07^{2}}\\ & =196 \end{array}$$

Where,

n = minimum required sample size
Z = 1.96 at 95% Confidence Interval (CI)
p = prevalence taken as 50% for maximum sample size calculation
q = 1-p
e = margin of error, 7%

The minimum required sample size was 196. However, 208 participants were included in the study.

A semi-structured questionnaire was developed. The questionnaire had two parts; the first part consisted of socio-demographic information and obstetric history. The second part consisted of various questions that assessed the uses of contraception. Information on the use of any form of contraceptive methods that women were using currently for any duration was taken. Pretesting was done for the questionnaire and modifications were done accordingly. The questionnaires were directly administered by investigators and research volunteers in a dedicated place in the OPD maintaining adequate privacy and confidentiality.

Data was collected and analysed using Microsoft Excel 2013. Point estimate and 95% CI were calculated.

## RESULTS

Out of 208 patients, contraceptive use was found in 146 (70.19%) (63.97-76.41, 95% CI) women. The mean year of use of contraception was 4.98±5.42 years. Short-acting reversible contraception (SARC) was used by 97 (66.44%). The most commonly used contraceptive device was Depo Provera 43 (29.45%) followed by male barrier methods 29 (19.86%) (Table 1).

**Table 1:** Types of contraceptive devices used (n= 146).

Type of contraceptives devices	n (%)
SARC	97 (66.44)
Depo provera	43 (29.45)
Male barrier method (condom)	29 (19.86)
Oral contraceptive pills (OCPs)	25 (17.12)
Long-acting reversible contraception (LARC)	23 (15.75)
Implants	14 (9.59)
Intra-uterine contraceptive devices (IUCD)	9 (6.16)
Permanent sterilisation	21 (14.38)
Tubal ligation	13 (8.90)
Vasectomy	8 (5.48)
Emergency contraceptive pills	5 (3.42)

Hospitals, health posts and health professionals were the most common source of information about contraception by 59 (40.41%) (Table 2).

**Table 2:** Information about contraception (n= 146).

Source	n (%)
**Source of information about contraception**	
Hospitals/health professionals/health post	59 (40.41)
Television/Radio	37 (25.34)
Female community health volunteers	19 (13.01)
Friends and family	17 (11.64)
Social media	14 (9.59)
**Who suggested you use contraceptives?**	
Health professional/hospital/health post	66 (45.21)
Husband/Partner	47 (32.19)
Female community health volunteers	19 (13.01)
Friends and family	11 (7.53)
Pharmacist	3 (2.05)
**Source of Contraceptive**	
Local medical shop/pharmacy	58 (39.73)
Hospital	54 (36.99)
Local health post/primary health Care	30 (20.55)
Private clinics	4 (2.74)

The mean age of the participants was 32.29±7.26 years (Range: 20 to 49). The majority 41 (29.10%) of the women were in the 26-30 years age group. Among them, 20 (13.70%) did not have any formal education.

A total of 69 (47.26%) were involved in agriculture and agriculture-related business (Table 3).

**Table 3:** Socio-demographic profile (n= 146).

Variables	n (%)
**Age (years)**	
20-25	25 (17.12)
26-30	41 (29.10)
31-35	34 (23.29)
36-40	24 (16.44)
41-45	16 (10.96)
46-49	6 (4.11)
**Religion**	
Hinduism	99 (67.81)
Buddhism	30 (20.55)
Christianity	13 (8.90)
Muslim	4 (2.74)
**Education level**	
No formal education	20 (13.70)
Primary	39 (26.71)
Secondary	28 (19.18)
Higher secondary	21 (14.38)
Bachelors	25 (17.12)
Masters and above	13 (8.90)
**Education level of husband**	
No formal education	10 (6.85)
Primary	43 (29.45)
Secondary	17 (11.64)
Higher secondary	34 (23.28)
Bachelors	29 (19.86)
Masters and above	13 (8.90)
**Occupation**	
Agriculture and agriculture-based business	69 (47.26)
Service	26 (17.80)
Housewife	24(16.44)
Business	19 (13.01)
Labor	8 (5.48)
**Occupation of husband**	
Service	58 (39.73)
Agriculture and agriculture-based business	38 (26.03)
Business	25 (17.12)
Labor	16 (10.96)
Foreign based job	9 (6.16)

A total of 132 (90.41%) were multiparous and 51 (34.93%) participants had at least one abortion. Among all the abortions, 37 (25.34%) underwent a medical abortion, 18 (23.08%) underwent surgical abortion and 23 (29.49%) underwent a spontaneous abortion. The majority of them were done for 46 (83.64%) unwanted pregnancies followed by 5 (9.09%) missed abortions, 3 (5.45%) for molar pregnancy, and 1 (1.82%) for severe maternal depression (Table 4).

**Table 4:** Obstetric history profile of participants (n= 146).

Parameters	n (%)
**Parity**	
Nulliparity	14 (9.59)
Multiparity	132 (90.41)
**Abortion**	
Total women who had previous abortions	51 (34.93)
One	30 (20.55)
Two	15 (10.27)
Three	6 (4.11)
Total abortion events	78 (53.42)

A total of 9 (4.37%) had never used any form of contraceptive devices and 53 (25.48%) had discontinued using contraceptive devices. The most common reason for discontinuation of contraceptive devices was to conceive 25 (47.17%) (Table 5).

**Table 5:** Reason for discontinuation of contraceptive devices (n= 146).

Reasons for discontinuation	n (%)
To conceive	25 (47.17)
Due to side effects	12 (22.64)
Husband abroad/not living together	9 (16.98)
Using natural methods of contraception	4 (7.55)
Sexually not active (divorced/widowed)	2 (3.77)

## DISCUSSION

In our study, 70.19% women were currently using some form of contraceptive devices while in other studies it was 44.39%, 66.3%, 73.75%, 85.5%, and 92%.^1,7-10^ This implies that in our study comparatively a lesser number of participants are using contraceptive devices.

In our study, the most commonly used contraceptive device was injectable, similarly, in another study too the most common contraceptive devices were injectable.^1,10^ while in other studies, the most common contraceptive devices were OCPs and condoms.^1,4,11^ We can conclude from this information that the choice of contraceptive devices can vary among different study populations and study sites.

Depo provera was most commonly used in our study, by about 30% while in other studies it was used among 2.6%,^9^ 26.7%,^12^ 29.3%,^11^ 37.4%,^10^ and 54.7%.^1^ Condoms were the second most used method, used by about 20% while in other studies it ranged from 19.15%^9^ to 37.1%.^11^ Similarly, OCPs were used by 17% of participants while their use was about 20% in other studies.^9,10^

Regarding the permanent method of contraception, tubal ligation was done by about 9% of patients, similar to our study, it was 11.11 %^9^ and 12.7%^12^ in other studies. Vasectomy was practised by about 5.5% of participants in our study while it was found to be practised by 10.4% in another study done in Nepal.^9^ Our finding of female sterilisation was found to be comparable in other studies but participants using vasectomy were found to be lesser.

The source of information was by radio (79%) and friends (60.7%) while health institutions were 11% and health workers were 60%.^1^ But in our study radio/ television was the source of information for 25.34% and friends/family in 11.64% while health institutes and health professionals were sources of information for 40.42%. In a study done in Northern India, the most common source of information on contraception was media in 55.7%^4^ and 45%.^13^ In another study done in Pakistan, the main source of information were friends and families followed by health workers.^14^ In our study apart from radio/television, social media was the source of information for only 9.59%.

In our study, about 14% of participants had no formal education while in other studies 23% to 42% had no formal education.^1,12,10^ Similar to our study, the majority of participants had completed primary or secondary-level education.^1,10,12^ In contrast to our study where 27% of participants had a bachelor's or above level of education which was present in only 2.4%^10^ and 4%.^12^

In our study, most of the participants, about 47%, were engaged in agriculture or agriculture-based businesses. While in other studies 30%^10^ to 70%^12^ of the participants were engaged in agriculture About 16% of women were housewives in our study which is much less when compared to 78%.^1^ In our study about 18% was involved in services while in other studies it was found to be 12.7%^12^ and 28%.^10^ Similarly, 13% of participants were involved in business in our study which was about 16% in other studies.^10,12^

Globally, the majority of abortions are still the direct consequence of the non-use of any contraception. More than 90% of abortions are performed on unintended pregnancies. A total of 70% of unintended pregnancies are due to the non-use of contraception.^15^ In our study among the women with prior medical or surgical abortions, 83.64% were for unwanted pregnancies, which is quite high. This could be because they may have used abortion in substitution for contraception and it also reflects that the level of knowledge, attitude and practice of contraception is still suboptimal.

The major limitation of our study is that it is a single-centre study with a relatively small sample size. Therefore, the result of this study may not be generalisable in community settings and other institutional settings.

## CONCLUSIONS

The prevalence of contraception use is lower than in other studies done in similar settings. Contraception promotion programs have to be encouraged in order to raise awareness about different contraceptive devices and various aspects of them. This will promote good attitudes and efficient use of contraception. And as a result, it will decrease unwanted pregnancies hence the number of abortions and abortion-related complications.
